# Supplementary material for: Multi-decadal increase of forest burned area in Australia is linked to climate change
Source: Nat Commun. 2021 Nov 26;12:6921. doi: 10.1038/s41467-021-27225-4 (PMC8626427; doi:10.1038/s41467-021-27225-4)
Supplement: Supplementary file 1 — Supplementary Information [file 41467_2021_27225_MOESM1_ESM.docx]

Supplementary Information for

Multi-decadal increase of forest burned area in Australia is linked to climate change

Josep G. Canadell^1*^, C.P. (Mick) Meyer^2^, Garry Cook^3^, Andrew Dowdy^4^, Peter R. Briggs^1^, Jürgen Knauer^1^, Acacia Pepler^4^, Vanessa Haverd^1^

^1^Climate Science Centre, CSIRO Oceans and Atmosphere, Canberra, ACT 2601, Australia

^2^Climate Science Centre, CSIRO Oceans and Atmosphere, Aspendale, VIC 3195, Australia

^3^CSIRO Land and Water, CSIRO Land and Water, PMB 44, Winnellie, NT 0822, Australia

^4^Bureau of Meteorology, Climate Research Section, Bureau of Meteorology, Melbourne, Australia

*Corresponding author

[pep.canadell@csiro.au](mailto:pep.canadell@csiro.au)

Supplementary Figure 1. Comparison of wildfire burned area (km^2^) based on State and Territory agencies and NOAA-AVHRR-Landgate for 1988-2019 fire years, and NASA-MODIS for the 2002-2019 fire years (fire year: July to June).

Supplementary Figure 2. Seasonal trends of burned area in km^2^. All trends are statistically significant (p-value < 0.001) except for summer (p-value = 0.07). Autumn: March, April, May; Winter: June, July, August; Spring: September, October, November; Summer: December, January, February. Data: AVHRR-Landgate (1988-2019).

Supplementary Figure 3. Bi-monthly trends of burned area in km^2^. Data: AVHRR-Landgate (1988-2019).

Supplementary Figure 4. Burned area (km^2^) of wildfires and prescribed burns for 1960 to 2019 fire year by States and Territories. NSW+ACT: New South Wales and Australian Capital Territory; VIC: Victoria; WA: Western Australia; QLD: Queensland; TAS: Tasmania; SA: South Australia. Data: State and Territory agencies (1960-2018) and MODIS (2019).

Supplementary Figure 5. Same as Figure 4 in main text but for the whole Australia. Number of years since the last fire (decadal mean) for the last four decades for forested areas. Data: State and Territory fire histories (1980-2018) and MODIS (2019).

Supplementary Figure 6. Trends in the number of days in which Forest Fire Danger Index (FFDI) meets or exceeds (a) 25, linear fit, or (b) 50, linear fit, over the fire years of 1979 to 2018 and averaged over forest ecosystems in Australia (Supplementary Table 1). Relationship between FFDI (c) ≥25 (exponential fit) and (d) ≥50 (exponential fit) and burned area for the fire years of 1988 to 2018 and 1988-2019 fire years (Supplementary Table 1). Data: AVHRR-Landgate (1988-2019). Triangle shows 2019 fire year. As in Figure 6 in main text but c) and d) with exponential fits.

Supplementary Figure 7. Linear relationships between climate and fuel variables, fire years (July to June). Triangle shows 2019 fire year (not available for lightning). Data: AVHRR-Landgate (1988-2019).

Supplementary Figure 8. Spatial distribution of burned area (km^2^) for 1988-2018 using a) NOAA-AVHRR Landgate data, and b) States and Territories agency data.


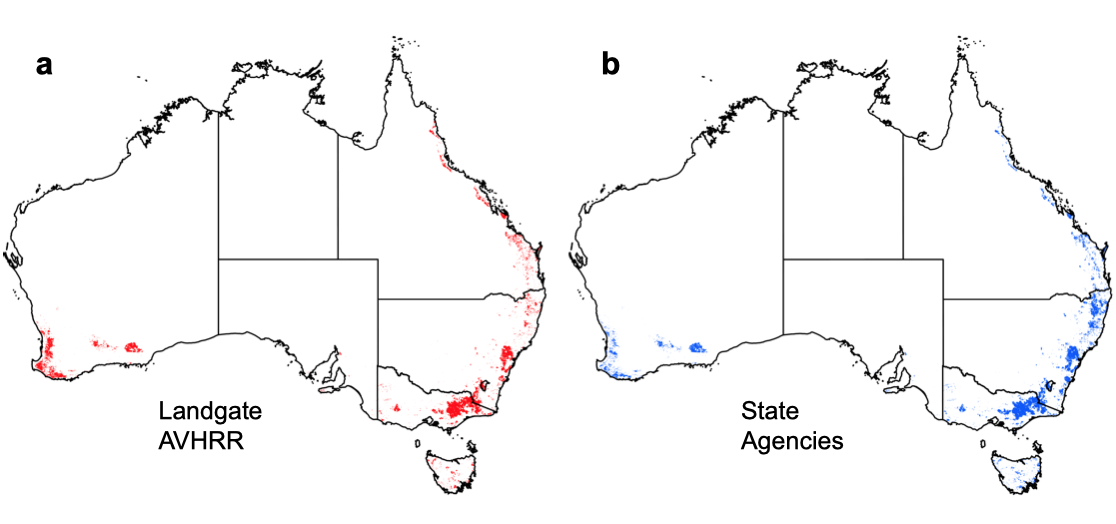


Supplementary Table 1. Regression parameters of linear (Y = a + bx) and exponential relationships (Y = a e^bx^) between burned area and the forest fire danger index (FFDI) with time, and of the relationships of burned area in forest ecosystems with days of very high (≥25) and severe (≥50) forest fire danger (FFDI) per fire year. Regressions that exclude 2019 fire year are to show the historical trends before the unprecedented 2019 burned area. Data: AVHRR-Landgate (1988-2019).

| **No.** | **Dependent variable** | **Independent variable** | **Relationship** | **a (SE)** | **b (SE)** | **R^2^** |
| --- | --- | --- | --- | --- | --- | --- |
| 1 | Burned area – all Australia  (‘000 km^2^) | Fire year  1988 – 2018 (x = t - 1987) | Linear | 285.8 (78.5) | 10.2 (4.3) | 0.164  (p = 0.024) |
| 2 | Burned area – all Australia  (‘000 km^2^) | Fire year  1988 – 2019 (x = t - 1987) | Linear | 302.7 (77.8) | 8.7 (4.1) | 0.129  (p = 0.044) |
| 3 | Burned area –forests (‘000 km^2^) | Fire year  1988 – 2018 (x = t – 1987) | Linear | 1.29 (1.56) | 0.174 (0.085) | 0.126  (p = 0.050) |
| 4 | Burned area –forests (‘000 km^2^) | Fire year  1988 – 2019 (x = t – 1987) | Linear | -2.06 (3.64) | 0.478 (0.193) | 0.170 (p=0.019) |
| 5 | Burned area – forests (‘000 km^2^) | Fire year  1988 – 2018 (excl 2002, 06) (x = t – 1987) | Linear | 0.43 (0.70) | 0.170 (0.038) | 0.424  (p < 0.001) |
| 6 | Burned area –forests (Autumn / Winter)  (km^2^) | Fire year  1988 – 2018 (x = t – 1987) | Exponential | 82.1 (40.0) | 0.088 (0.018) | 0.566  (p <0.001) |
| 7 | Burned area –forests (Autumn / Winter)  (km^2^) | Fire year  1988 – 2019 (x = t – 1987) | Exponential | 33.0 (22.3) | 0.127 (0.023) | 0.652  (p <0.001) |
| 8 | Burned area –forests (‘000 km^2^) | Days with FFDI > 25 (1988 – 2018) | Exponential | 0.254 (0.111) | 0.194 (0.022) | 0.754  (p < 0.001) |
| 9 | Burned area –forests (‘000 km^2^) | Days with FFDI > 25 (1988 – 2019) | Exponential | 0.670 (0.122) | 0.138 (0.006) | 0.951  (p < 0.001) |
| 10 | Burned area –forests (‘000 km^2^) | Days with FFDI > 50 (1988 – 2018) | Exponential | 0.802 (0.172) | 1.58 (0.12) | 0.833  (p < 0.001) |
| 11 | Burned area –forests (‘000 km^2^) | Days with FFDI > 50 (1988 – 2019) | Exponential | 1.90 (0.27) | 0.909 (0.041) | 0.942  (p < 0.001) |
| 12 | Days with FFDI > 25 - forests | Fire year (1979 – 2018) | Linear | -302.0 (114.0) | 0.157 (0.057) | 0.166  (p = 0.009) |
| 13 | Days with FFDI > 50 - forests | Fire year (1979 – 2018) | Linear | -31.6 (12.2) | 0.0162 (0.0061) | 0.156  (p = 0.012) |

Supplementary Table 2. Number of years since the last fire, YSLF (decadal mean±standard deviation) by States and Territories for the last four decades for forested areas that have burned at least once since fire records began. Data: State and Territory fire histories.

| Decade | NSW | | VIC | | QLD | | SA | | WA | | TAS | | ACT | |
| --- | --- | --- | --- | --- | --- | --- | --- | --- | --- | --- | --- | --- | --- | --- |
|  | Mn | SD | Mn | SD | Mn | SD | Mn | SD | Mn | SD | Mn | SD | Mn | SD |
| 1980s | 69 | 1 | 56 | 2 | 112 | 2 | 58 | 18 | 73 | 0 | 81 | 2 | 73 | 8 |
| 1990s | 66 | 1 | 61 | 2 | 113 | 1 | 48 | 3 | 57 | 7 | 80 | 2 | 77 | 3 |
| 2000s | 49 | 6 | 47 | 12 | 94 | 14 | 53 | 1 | 46 | 3 | 69 | 9 | 25 | 31 |
| 2010s | 44 | 12 | 34 | 4 | 47 | 21 | 45 | 9 | 36 | 4 | 44 | 14 | 18 | 4 |

Supplementary Table 3. Parameters of multivariate regressions between logarithmic of log (burned area) predicted by climate related fire risk factors: FFDI, C-Haines, and dry lightning. The variables are ordered from high to low explanatory power, and the lowest is dropped in the next cell down. Lower Akaike Information Criterion (AIC) means better model performance relative to its complexity (number of parameters). Statistical significance (0 ***, 0.001 **, 0.01 *, 0.05 “.”). 3a) FFDI25, and 3b) FFDI50.

| 3a |  | | | 3b |  | | |
| --- | --- | --- | --- | --- | --- | --- | --- |
| Explanatory  variables | R^2^ | R^2^ adj. | AIC | Explanatory  variables | R^2^ | R^2^ adj. | AIC |
| FFDI25 **  C-Haines  Dry lightning . | 0.67 | 0.63 | 62.1 | FFDI50 ***  C-Haines  Dry lightning . | 0.74 | 0.71 | 55.1 |
| FFDI25 ***  C-Haines | 0.69 | 0.66 | 71.2 | FFDI50 ***  Dry lightning . | 0.71 | 0.69 | 56.4 |
| FFDI25 ***  Dry lightning . | 0.64 | 0.62 | 62.2 | FFDI50 ***  C-Haines | 0.70 | 0.68 | 75.4 |
| FFDI25 *** | 0.68 | 0.67 | 70.2 | FFDI50 *** | 0.70 | 0.69 | 67.8 |
| C-Haines *** | 0.40 | 0.38 | 89.8 |  | | | |
| Dry lightning *** | 0.39 | 0.36 | 75.4 |  |  |  |  |

Supplementary Table 4. Regression parameters of linear (Y = a + bx) relationships of CABLE aboveground biomass and litter over time as shown in Figure 8.

|  | Dependent variable | Independent variable | Relationship | a | b (SE) | Correlation coefficient |
| --- | --- | --- | --- | --- | --- | --- |
| 1 | Aboveground woody biomass | Calendar years | Linear | 6.77 | 0.005091 (0.00141) | p value: 0.0006211 |
| 2 | Leaf biomass | Calendar years | Linear | 0.72 | 0.000199 (7.86e-05) | p value: 0.01418 |
| 3 | Coarse woody debris | Calendar years | Linear | 0.51 | 0.000329  (0.000524) | p value: 0.5335 |
| 4 | Fine litter (structural) | Calendar years | Linear | 2.0 | -0.0006249 (6.93e-05) | p value:  1.2e-12 |
| 5 | Very fine litter (metabolic) | Calendar years | Linear | 0.07266 | -2.186e-05 (1.095e-05) | p value: 0.05058 |

Supplementary Table 5. Data sources for the fire histories of burned area and ancillary information from Australian State and Territory agencies.

| **No** | **State** | **Agency** | **Database** | **URL** |
| --- | --- | --- | --- | --- |
| 1 | New South Wales | Department of Planning, Industry and Environment | NPWS Fire History - Wildfires and Prescribed Burns | <https://datasets.seed.nsw.gov.au/dataset/fire-history-wildfires-and-prescribed-burns-1e8b6> |
| 2 | Victoria | Department of Environment, Land, Water & Planning | Fire History Records of Fires primarily on Public Land | <https://discover.data.vic.gov.au/dataset/fire-history-records-of-fires-primarily-on-public-land> |
| 3 | South Australia | Department for Environment and Water | Bushfires and Prescribed Burns History | <https://data.sa.gov.au/data/dataset/fire-history> |
| 4 | Western Australia | Department of Biodiversity, Conservation and Attractions | DBCA Fire History (DBCA-060) | <https://catalogue.data.wa.gov.au/dataset/dbca-fire-history> |
| 5 | Tasmania | Department of Primary Industries, Parks, Water and Environment | List Fire History – Statewide Coverage | <http://listdata.thelist.tas.gov.au/opendata/> |
| 6 | Queensland | Brisbane City Council | Wildfire History — SHP | <https://www.data.brisbane.qld.gov.au/data/dataset/wild-fire-history/resource/2e8c7996-b864-4166-a1a0-953370ab63e5?inner_span=True> |
| 7 | All states | Australian Government Department of Agriculture, Water and the Environment | National Indicative Aggregated Fire Extent Datasets (NIAFED) | <http://www.environment.gov.au/fed/catalog/search/resource/details.page?uuid=%7B9ACDCB09-0364-4FE8-9459-2A56C792C743%7D> |
